# Extreme variation in recombination rate and genetic diversity along the Sylvioidea neo‐sex chromosome

**DOI:** 10.1111/mec.16532

**Published:** 2022-06-06

**Authors:** Suvi Ponnikas, Hanna Sigeman, Max Lundberg, Bengt Hansson

**Affiliations:** ^1^ Department of Biology Lund University Lund Sweden; ^2^ Ecology and Genetics Research Unit University of Oulu Oulu Finland

**Keywords:** evolutionary rates, genetic variation, great reed warbler, linkage map, neo‐sex chromosome, recombination

## Abstract

Recombination strongly impacts sequence evolution by affecting the extent of linkage and the efficiency of selection. Here, we study recombination over the Z chromosome in great reed warblers (*Acrocephalus arundinaceus*) using pedigree‐based linkage mapping. This species has extended Z and W chromosomes (“neo‐sex chromosomes”) formed by a fusion between a part of chromosome 4A and the ancestral sex chromosomes, which provides a unique opportunity to assess recombination and sequence evolution in sex‐linked regions of different ages. We assembled an 87.54 Mbp and 90.19 cM large Z with a small pseudoautosomal region (0.89 Mbp) at one end and the fused Chr4A‐part at the other end of the chromosome. A prominent feature in our data was an extreme variation in male recombination rate along Z with high values at both chromosome ends, but an apparent lack of recombination over a substantial central section, covering 78% of the chromosome. The nonrecombining region showed a drastic loss of genetic diversity and accumulation of repeats compared to the recombining parts. Thus, our data emphasize a key role of recombination in affecting local levels of polymorphism. Nonetheless, the evolutionary rate of genes (dN/dS) did not differ between high and low recombining regions, suggesting that the efficiency of selection on protein‐coding sequences can be maintained also at very low levels of recombination. Finally, the Chr4A‐derived part showed a similar recombination rate as the part of the ancestral Z that did recombine, but its sequence characteristics reflected both its previous autosomal, and current Z‐linked, recombination patterns.

## 1 INTRODUCTION

Recombination plays a key role in evolution and adaptation by rearranging alleles into new haplotypes, thereby creating novel genetic variation that selection can act upon, and by breaking the linkage between loci allowing them to evolve independently. That recombination is critical for maintaining the fitness of large genomic regions, becomes evident when recombination is lacking (Charlesworth & Charlesworth, 2000). Without recombination, selection operates on the level of entire linked regions (instead of on more independently segregating single mutations), which lowers the fixation probability of beneficial mutations and the efficiency by which mildly deleterious mutations are being purged. Indeed, the outcome of selection on any genomic region is a composite result of selection acting on many individual variants, and the multitude of variants involved depends on the recombination rate in that region. The population genetic processes linking a lack (or low level) of recombination with lowered adaptative potential are often referred to as Muller's ratchet and Hill‐Robertson interferences (Hill & Robertson, 1966; Muller, 1964). The former refers to stochastic loss of mutation‐free sequence, leading to the accumulation of deleterious mutations. The latter refers to processes where linkage between sites under selection reduces the overall effectiveness of selection. The end results of these processes, such as loss of genetic variation, accumulation of repeats and ultimately sequence degeneration, are often observed on heteromorphic sex chromosomes and other regions where recombination stopped a long time ago (Charlesworth & Charlesworth, 2000).

In addition of having strong impact on sequence evolution, recombination can also be under selection itself: depending on the signs and strengths of epistatic fitness interactions between linked genes, recombination can be either selectively beneficial by breaking unfavourable combinations or harmful by breaking coadapted gene complexes (Butlin, 2005). Recombination rates have been observed to vary between species, populations, sexes, and individuals (Stapley et al., 2017a). Recombination is also not uniform within the genome, which is reflected by recombination hotspots, where the rate is much higher than average. These regions can be both stable and dynamic, depending on the taxa and molecular mechanism regulating the recombination events (Sardell & Kirkpatrick, 2020; Stapley et al., 2017b). The rate of recombination further depends on the physical position along the chromosome, with centromeric parts often showing lower rates compared to chromosome ends. This bias in recombination towards telomeres occurs especially in males in vertebrates, while females show more evenly distributed patterns (Sardell & Kirkpatrick, 2020).

Heteromorphic sex chromosomes stand out from the general recombination patterns at the genome level. Their evolution is dictated by the lack of recombination between large sections of the sex chromosome pair in the heterogametic sex (XY males and ZW females, in male and female heterogametic systems, respectively), while recombination proceeds in the homogametic sex (XX females and ZZ males). This phenomenon is observed across a wide range of species including animals, plants and fungi (Bachtrog et al., 2014). Many selective forces have been suggested to drive the observed recombination suppression, including sexual antagonism, heterozygote advantage and meiotic drive, of which the first one is the best supported by theory (Ponnikas et al., 2018). Even though recombination suppression is often expanding along the sex chromosomes over time, sex chromosomes also tend to retain recombining region(s), known as the pseudoautosomal region (PAR). The persistence of PARs could be due to selection for maintaining recombination or because these regions are essential for the proper segregation of sex chromosomes during meiosis which involves chiasma formation (Otto et al., 2011).

Birds have a female heterogametic sex determination system (ZW females and ZZ males), where females in the vast majority of species do not recombine for most parts of their sex chromosome pair and consequently exhibit a small PAR. There are, however, exceptions, such as in some species of Palaeognathae (e.g., the common ostrich, Struthio camelus, Yazdi & Ellegren, 2018), which retain recombination over a substantial part of the sex chromosomes and thus have a large PAR. Also, while the avian sex chromosomes have a deep ancestral homology dating back approximately 100 million years (Myr), a few lineages have neo‐sex chromosomes formed by fusions between the ancestral sex chromosome pair and (parts of) one or more autosomes (Dierickx et al., 2020; Gan et al., 2019; Huang et al., 2021; Kretschmer et al., 2021; Pala et al., 2012; Sigeman et al., 2019, 2020). In general, neo‐sex chromosome formation is predicted to be favoured when previously autosomal genes gain advantages by becoming sex‐linked and this could be mediated by sexually antagonistic selection or, in inbreeding populations, by heterozygote advantage (Charlesworth & Charlesworth, 1980; Charlesworth & Wall, 1999). One of the avian neo‐sex chromosome pairs occurs in the songbird superfamily Sylvioidea and was formed approximately 24 Myr ago when a part of autosome 4A (0–9.6 Mbp) was translocated to the ancestral sex chromosomes (both Z and W; Pala et al., 2012; Sigeman et al., 2021). The great reed warbler (*Acrocephalus arundinaceus*) belongs to Sylvioidea and earlier studies have presented an incomplete linkage map for the species based on microsatellites and AFLP, including the Z chromosome (Åkesson et al., 2007; Hansson et al., 2005). Although, the fine‐scale recombination pattern across the great reed warbler genome remains largely unknown due to the low marker‐density of these linkage maps, the earlier maps strongly suggest that males recombine less than females on the autosomes and that both sexes have markedly shorter map distances compared to other bird species, such as chicken (*Gallus gallus*, Groenen et al., 2009) and collared flycatcher (*Ficedula albicollis*, Kawakami et al., 2014). For example, Dawson et al. (2007) observed linkage map distances of male and female great reed warblers to be only 6.3 and 13.3% of those of the chicken, respectively.

These earlier findings of a relatively low level of recombination in the great reed warbler, especially in the homogametic males, together with the fact that the sex chromosome pair does not recombine outside the small PAR in the heterogametic females, suggest very low recombination rates along the species' Z chromosome. This may generate relatively large evolutionary units of cosegregating loci for selection and drift to act upon, which would in turn affect the efficiency of selection and amount of genetic diversity in the great reed warbler sex chromosomes. Thus, studying the rate and pattern of recombination in relation to sequence polymorphism in the great reed warbler can help to evaluate and understand the causes and consequences of recombination for sequence and genome evolution.

A draft genome assembly is available for the great reed warbler in which 22 scaffolds have been assigned to the Z chromosome using male‐to‐female coverage ratio and heterozygosity differences of resequenced individuals (Sigeman et al., 2021). However, the physical order of the scaffolds is still lacking. Here, we use linkage mapping in a multigenerational pedigree to order and orient the entire Z chromosome of this species. We describe the recombination landscape along this chromosome and evaluate how the recombination rate varies depending on the chromosome position (central vs. telomeric parts). Then, we analyse the association between recombination rate and genetic diversity, linkage disequilibrium and evolutionary rate (measured by dN/dS). The autosome–sex chromosome fusion forming the neo‐Z chromosome (and neo‐W chromosome) in our study species provides an opportunity to study the temporal aspect of the causes and consequences of recombination, since the fused part of autosomal origin has been sex‐linked for a much shorter time compared to the ancestral Z (referred to as added‐ and ancestral‐Z, respectively, from now on).

## 2 MATERIALS AND METHODS

### 2.1 Pedigree and DNA samples

We used multigeneration pedigree data from a long‐term study population of great reed warblers at Lake Kvismaren in Sweden. The study species' ecology, as well as monitoring and sampling practices, are described in Bensch et al. (1998), Hansson et al. (2005, 2018) and Åkesson et al. (2007). The pedigree was collected between the years 1984 and 2004 (see Hansson et al., 2018). Samples were collected nondestructively from the birds and with the appropriate permissions from the Malmö‐Lunds djurförsöksetiska nämnd (M 45–14 and 17,277–18). DNA was extracted from these field‐collected blood samples using a standard phenol/chloroform protocol. After all filtering steps (see below), the final data consisted of 240 unique individuals (146 offspring) of which 221 individuals were genotyped (raw sequence reads are deposited in NCBI BioProject PRJNA445660, Hansson et al., 2018). Note that several of the 240 individuals appear several times in the pedigree (as offspring and as parent one or more times).

### 2.2 Genomic data

Single digest (Sbf1) restriction site‐associated DNA (RAD) library construction, sequencing (Illumina 100 bp paired‐end), trimming and demultiplexing was performed by BGI (Hong Kong; see Hansson et al., 2018). The sequence reads were mapped to the female great reed warbler reference genome (Sigeman et al., 2021) with the MEM algorithm (Li, 2013) in BWA v. 0.7.15 (with default settings except for the –M flag to mark shorter split reads as secondary to ensure downstream compatibility with Picard tools). The output was converted to BAM format and sorted with SAMtools 1.4 (Li et al., 2009). Duplicate reads were removed with Picard tools v. 2.6 (at http://broadinstitute.github.io/picard) and the remaining reads were realigned with GATK 3.8–0 (McKenna et al., 2010).

To find high‐confidence genetic markers, we called and filtered single nucleotide polymorphisms (SNPs) with three different pipelines (Supporting Information 1) and then included only those SNPs in the final data set that were found in all three filtered outputs. As erroneous SNPs can cause map inflation and incorrect ordering of markers, we aimed for as error free data as possible. After filtering, 50,265 markers on 3005 scaffolds (covering 1,200,165,392 bp) remained. Ten of these scaffolds had previously been identified as Z‐linked based on their sex‐specific coverage and heterozygosity in analyses of whole‐genome resequenced females and males, and synteny to the zebra finch Z chromosome (Sigeman et al., 2021).

We checked for Mendelian inheritance consistency in the pedigree individuals with VIPER (Paterson et al., 2012) using a sample of 10,000 loci, which showed less than 6% genotype error in the majority of individuals, with the exception of 10 birds with higher genotype errors (6–18%) suggesting incorrect pedigree relationships. We checked the parentage for these birds in CERVUS v. 3.0.7 (Kalinowski et al., 2007), which corrected the pedigree position for five of them. The remaining five birds with uncertain parentage were removed from the data.

### 2.3 Linkage map for Z: Anchoring and ordering scaffolds

A linkage map was built with Lep‐MAP3 (Rastas, 2017, see Supporting Information 2 for the scripts used in the linkage mapping). For these analyses the pedigree data were divided into 79 three‐generation families (2–4 grandparents, 2 parents and varying number of offspring). As some individuals were present in multiple (different) families (e.g., as offspring, parent or grandparent), the pedigree data consisted of 511 nonunique individuals. The ParentCall2 module in Lep‐MAP3 identified a high proportion of Z‐linked markers (being hemizygous in females) on seven scaffolds (scaffolds 2, 5, 31, 94, 98, 134, 4139), all of which had been identified as Z‐linked in Sigeman et al. (2021). Moreover, our Lep‐MAP3 analysis identified one additional Z‐linked scaffold that the previous analysis failed to detect (scaffold 92, size 203,298 bp).

To localize the border between the sex‐linked part of Z and the PAR, we calculated sex‐specific heterozygosity and coverage for all Z‐linked SNPs in VCFtools v. 0.1.14 (Danecek et al., 2011) using the pedigree birds (Figure S1). Eight SNPs showed heterozygosity and coverage patterns in line with a scenario where the W copy had been mapped to the Z (both F/M coverage and female heterozygosity close to 1). We excluded these SNPs from further Lep‐MAP3 steps, as they could interfere with the linkage mapping. We also removed markers within sex‐linked Z‐scaffolds (identified with ~0.5 F/M coverage ratio) that were not recognized as sex‐linked by Lep‐MAP3. This was a conservative approach as we expect all Z‐linked SNPs to be invariant (hemizygous) in females due to the loss of or deep divergence to the orthologous regions on the W. The remaining number of markers was 50,257 overall and 850 on the eight Z‐scaffolds.

We used the module SeparateChromosomes2 with LOD score 16 to form linkage groups (LGs). We chose this LOD score since it resulted in defined LGs with synteny to single chromosomes in zebra finch genome assembly (Warren et al., 2010) with one exception: the known fusion between chromosomes Z and 4A. Lower LOD scores generally resulted in LGs that included (based on zebra finch synteny) multiple chromosomes; thereby giving the impression of several fusions. Conversely, higher LOD scores split chromosomes into many LGs (e.g., due to physical gaps in marker distributions) giving the impression of several fissions in our study species. At LOD 16 the sex‐linked markers were initially assigned to two LGs, but with slightly lower LOD score (15) these SNPs formed a single LG. Thus, the division into two LGs was concluded to be artificial and we combined all Z‐markers into one LG for the further steps.

Additional markers were assigned to LGs in the JoinSingles2 module, by lowering the LOD score from 15 to 3 while simultaneously controlling the quality of linkage with lodDiff‐setting, which prevents the markers from assigning to more than one LG with equally high likelihoods. The last step of assigning new markers (LOD = 3 and lodDiff = 2) was repeated twice. We ran ten marker orderings (starting from random order each time) in the module OrderMarkers2 and chose the order with the highest likelihood. We used the Kosambi mapping function, utilized grandparent genotypes to phase the data and set recombination to occur only in males (recombination2 = 0). The latter setting applies to most of the Z but is not optimal for the PAR region where both sexes recombine. To be certain that this setting did not interfere with the marker ordering and the following anchoring of the scaffolds (see below), we also ran the marker ordering and anchoring without the assumed PAR scaffold (Supporting Information 3).

Since the recombination varies between sex‐linked Z and PAR (only males recombine in the former and both sexes in the latter), we also ran the marker ordering step separately for the PAR (9 SNPs) to be able to use more suitable settings (i.e., allow both sexes to recombine) for this region. However, the OrderMarkers2 was unable to find the best marker order for the PAR as all 10 runs had identical likelihood values, but different marker orders. Therefore, we cannot present exact recombination rates for both sexes in this region and use marker order according to the physical map order along the scaffold.

Scaffold ordering and orientation was done in ALLMAPS (Tang et al., 2015, see Supporting Information 2 for scripts used in ALLMAPS) with default settings based on the male linkage map (best marker order) for the Z chromosome. The software added an arbitrary 100 bp gap between each of the scaffolds.

### 2.4 Recombination rate

To get the most accurate estimates on recombination rates, we re‐evaluated the genetic distances for the final physical order of markers (*N* = 664) based on their anchored (liftover) positions in the ALLMAPS output (Supporting Information 2). Genetic distances were re‐evaluated with the OrderMarkers2 module but the order itself was not evaluated further. Markers without anchored position in the final Z chromosome were excluded at this point. The local recombination rate along the chromosome was estimated with the MareyMap web‐service (http://lbbe‐shiny.univ‐lyon1.fr/MareyMapOnline/; Rezvoy et al., 2007; Siberchicot et al., 2015, 2017), where we fitted a LOESS function (span 0.2) to describe the relationship between the genetic and physical distances.

### 2.5 Recombination in relation to genome characteristics

We extracted the chromosomal region between the first and last markers in the male‐specific Z linkage map and divided this region into nonoverlapping 200 kbp bins (*N* = 427). The PAR region was also divided into 200 kbp bins (*N* = 4), but since we did not get accurate map distances for this interval, the PAR is included only in the comparisons between the recombination regions (see below). Bins overlapping with scaffold gaps were excluded from the analyses.

We used a fitted LOESS function to interpolate genetic distance point estimates for the start and end position of each bin within the male‐specific Z (i.e., excluding the PAR). The bin‐specific recombination estimate was then the genetic distance (cM) within these point estimates (Supporting Information 2).

We studied recombination in relation to several variables expected to be associated with it: genetic diversity (measured as the nucleotide diversity; *π*), linkage disequilibrium (r^2^ and D′), Tajima's D, Fay and Wu's H, proportion of exonic and repeat sequences, and GC content. These genome characteristics were calculated for each 200 kbp bin.

Linkage disequilibrium, genetic diversity and Tajima's D were estimated from five whole‐genome resequenced great reed warbler males (150 bp paired‐end Illumina Hiseq X) from the same population as the pedigree samples. For details regarding extraction, library preparation and trimming of raw reads, see Sigeman et al. (2021). Mapping of trimmed reads to the reference genome and subsequent removal of duplicated reads followed the same pipeline as used for the RAD sequencing libraries (see above). Variants were called on the Z chromosome using FreeBayes (Garrison & Marth, 2012) with default settings. The raw set of variants was filtered using a combination of VCFtools and vcflib (Garrison, 2012). The filtering steps included removal of variants overlapping annotated repeats or that had a mean coverage more than twice the median mean coverage of all variants. We further required that alternative alleles were supported by at least one read on each strand (SAF > 0 & SAR > 0) and by at least one read centred to the left and the right (RPL > 0 & RPL > 0), and that the quality of the variant should be more than 20. Genotypes with less than 10x coverage were recorded as missing and sites with more than 20% missing data were filtered. Finally, complex variants and multinucleotide polymorphisms (MNPs) were decomposed into separate SNPs and indels and biallelic SNPs were selected for downstream analyses. BCFtools (Li, 2011) was used to extract variants located in each window for which recombination rate had been estimated. For each window, VCFtools was used to calculate Tajima's D and nucleotide diversity. The latter can be biased if the number of callable sites varies a lot between windows, and, therefore, we calculated nucleotide diversity also by adjusting for the number of callable sites (see Figure S2; all results in relation to nucleotide diversity remained qualitatively the same with this adjustment and are thus not presented in the Results). To obtain measurements of linkage disequilibrium, we used Beagle version 5.1 (Browning & Browning, 2007) to phase and impute missing genotypes. For the phased data we used VCFtools to calculate pairwise r^2^ between SNPs that were at least 1 kbp apart within each window and only retained comparisons with r^2^ ≥ 0.1. For each window we calculated a mean r^2^ across all pairwise SNP comparisons. LD was estimated also with D′ for each window as it is insensitive to minor allele frequency. D′ was estimated in VCFtools where we calculated pairwise D′ values between variants at least 1 kb from each other in each window and calculated a window mean from the absolute values.

Fay and Wu's (2000) quantifies an excess of derived high‐frequency variants, which may be indicative of recent positive selection. To calculate this statistic, we first mapped short‐read sequence data of a male clamorous reed warbler (*Acrocephalus stentoreus*, NCBI BioProject PRJNA578893, sample ID: SRR16079857, Sigeman et al., 2021) using the same pipeline as for the great reed warbler samples. We next used FreeBayes to call genotypes for the positions where bi‐allelic variants had been detected in the great reed warbler males. The raw variants were filtered to obtain only genotypes with at least 10× coverage. The genotypes of the clamorous reed warbler were combined with the genotypes of the great reed warblers and from this combined data set we selected only variants for which the clamorous reed warbler was homozygous for either allele (after filtering 218,901 SNPs out of 246,191 remained, i.e., 89% of them). This data set was imported into PopGenome (Pfeifer et al., 2014), where the clamorous reed warbler was set as an outgroup to infer derived and ancestral alleles for computation of the H statistic.

Other genome variables (GC, exon and repeat proportions) were calculated from the annotated reference genome (GenBank accession: GCA_021534815.1; Sigeman et al., 2021). Exon and repeat proportions were calculated as the proportion of the bin that was covered by each sequence type in bedtools v. 2.26.0 (overlapping features were collapsed, Quinlan & Hall, 2010). The GC content was calculated with the pyfasta package (https://github.com/brentp/pyfasta/).

By utilizing the results of the recombination landscape, and sex‐specific coverage and diversity (from both resequencing and RAD‐seq data), we defined the border between the PAR and the sex‐linked Z and divided the Z chromosome into different recombination regions: PAR (where both sexes recombine), male‐recombining (MREC) and nonrecombining (NONREC; see Results). Then, we analysed the relationships between recombination and genome characteristics by comparing these three recombination regions, which should describe the effect of lacking recombination particularly. The relative amount of recombination between these regions is assumed to be: PAR > MREC > NONREC. Next, we studied the effect of the amount of recombination by correlations within the MREC region (together as well as separately for the ancestral‐ and added‐Z). The physical position effect on recombination was tested by correlation with the distance to chromosome end and by comparing chromosomal regions (ancestral‐ vs. added‐Z). All statistical tests were performed in R v. 1.3.1093 (R Core Team, 2021) and *p*‐values in region comparisons and recombination rate correlations were adjusted for multiple testing with Holm's (1979) correction.

### 2.6 Gene‐specific evolutionary rates

In order to assess the efficiency of selection in relation to recombination, we calculated gene‐specific evolutionary rates (dN, dS and dN/dS ratio) for the great reed warbler Z‐linked and PAR protein‐coding genes. We aligned the great reed warbler Z sequences together with orthologous CDS sequences from zebra finch and chicken, which were downloaded from the Ensembl BioMart database (Howe et al., 2021). Orthology information was obtained from the results in Sigeman et al. (2021). Of the original 470 genes, 396 genes had one‐to‐one orthologues from both these outgroups. We aligned these sequences using macse alignSequences v1.0.2 (Ranwez et al., 2011) (options ‐local_realign_init 1 ‐local_realign_dec 1). Identified frameshifts and stop codons were replaced with gaps using macse exportAlignment (options ‐codonForFinalStop ‐‐‐ ‐codonForInternalStop ‐‐‐ ‐codonForInternalFS ‐‐‐ ‐charForRemainingFS ‐). The sequences were converted from fasta to phylip format using the script SeqFormatConvert.seqFactory.SeqConverter from EasyCodeML v1.4 (Gao et al., 2019). We calculated branch‐specific dN, dS and dN/dS values for the three sequences using codeml from the PAML package v4.9 (Yang, 2007) using only sites without gaps (model = 1, NSsites = 0, cleandata = 1); a full control file is provided as Supporting Information 4). We further filtered genes with dS and/or dN/dS values higher than 2 and kept only genes with minimum length of 500 bp (cf. Sigeman et al., 2021). This left 332 genes for the analyses. The dN/dS ratio compares the amount of nonsynonymous substitutions at nonsynonymous sites (dN) to synonymous substitutions at synonymous sites (dS), and tests if a gene is under positive selection (dN/dS >1), purifying selection (dN/dS <1) or evolves neutrally (dN/dS = 1). The dN/dS rate is often observed to correlate negatively with recombination rate (Betancourt et al., 2009; Gossmann et al., 2011), which is thought to be caused by reduced efficiency of purifying selection on slightly deleterious mutations due to Hill‐Robertson interference in low recombination regions (Hill & Robertson, 1966). The recombination rate estimate for each gene's midpoint was inferred from the fitted LOESS function (using a span of 0.1, Supporting Information 2). The relationships between recombination and evolutionary rates were first studied by comparing the three recombination regions of Z and then by correlating the recombination and evolutionary rates within MREC. All statistical tests were performed in R v. 1.3.1093 and the *p*‐values in region comparisons and recombination rate correlations (separately for genes with and without W copy) were adjusted for multiple testing with Holm's (1979) correction.

## 3 RESULTS

### 3.1 Linkage map for Z: Anchoring and ordering scaffolds

The Z chromosome linkage group (LG‐Z) included 676 markers located on ten scaffolds. Eight of them were identified as Z‐linked in ParentCall2 (scaffolds 2, 5, 31, 92, 94, 98, 134 and 4139), while two scaffolds showed autosomal patterns regarding female/male read coverage and SNP heterozygosity (scaffolds 181 and 217), as would be expected for markers in the PAR. Scaffold 181 was excluded from LG‐Z after manual curation since it was assigned with only one marker (corresponding only to 0.1% of its total marker number) and showed synteny to chromosome 1A in zebra finch, great tit (*Parus major*) and collared flycatcher. Moreover, scaffold 98 showed a chimeric pattern indicative of a mis‐assembly. One part had markers assigned to LG‐Z and showed synteny to the fused part of chromosome 4A (i.e., added‐Z), while markers on the other part were left unassigned (i.e., none of the autosomally segregating SNPs on scaffold 98 were assigned) and showed synteny to chromosomes 19, 6 and 1 in the zebra finch. After this manual curation, the scaffold was split into one Z‐linked part (scaffold 98a; 1.17 Mbp) and one autosomal part (scaffold 98b; 0.36 Mbp), of which only 98a was included in LG‐Z (this also match the data in Sigeman et al., 2021). At this stage, scaffolds 2, 5, 31, 92, 94, 98a, 134, 217 (PAR) and 4139 remained.

**FIGURE 1:**
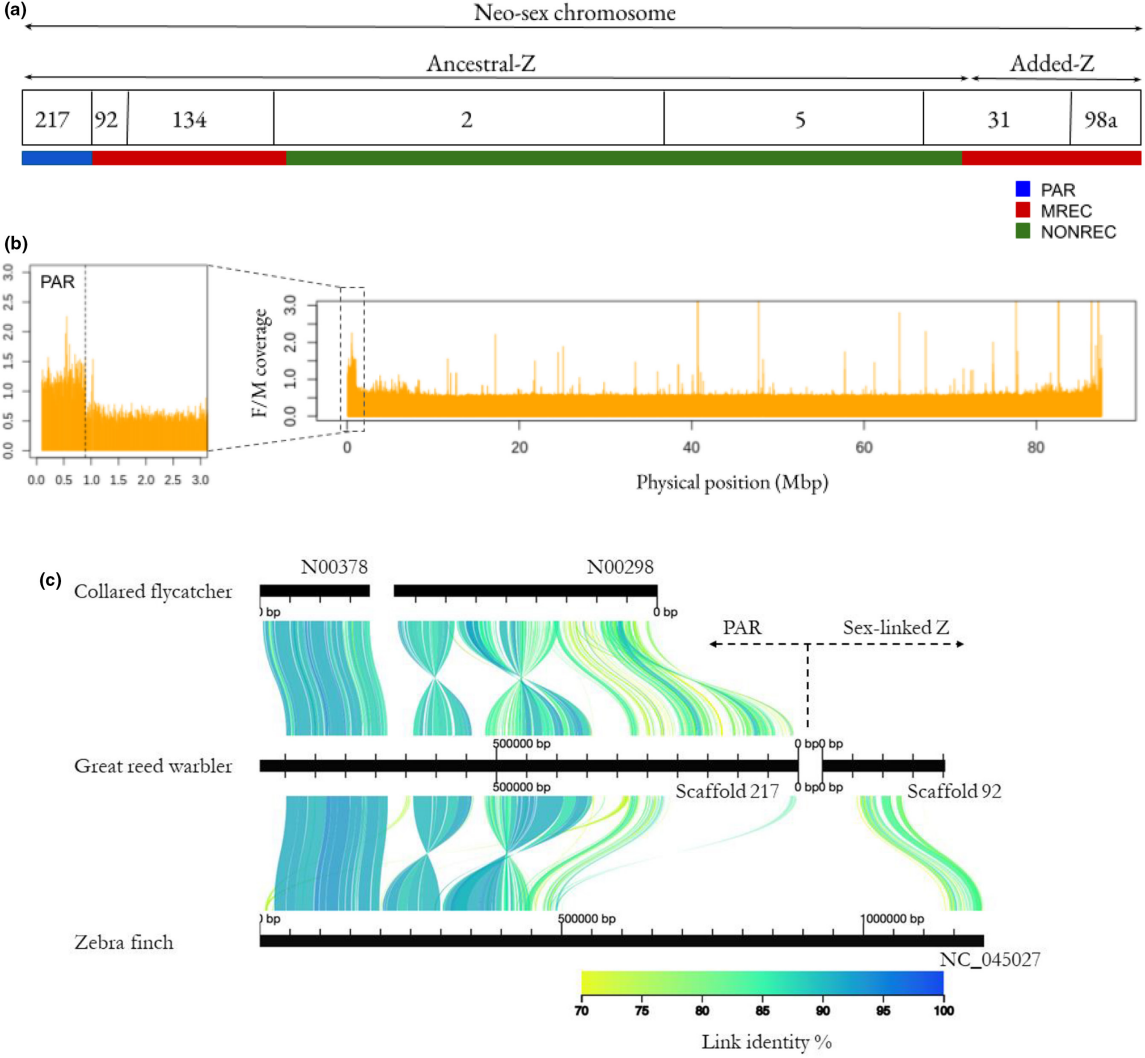
(a) A schematic illustration of the anchored great reed warbler Z chromosome scaffolds in relation to the three recombination regions: The pseudoautosomal region (PAR, where both sexes recombine), the male‐recombining regions at both ends (where only males recombine, MREC) and the non‐recombining region (no recombination in neither sex, NONREC). (b) Female‐to‐male coverage ratio (based on 10 re‐sequenced individuals) along the Z chromosome showing the border between the sex‐linked region and the PAR (latter zoomed in). (c) the PAR (scaffold 217) and the beginning of the sex‐linked region (scaffold 92) aligned to the previously identified flycatcher PAR scaffolds N00298 and N00378 (N02597 is not shown as it did not show synteny to our PAR) and the first 1.2 Mbp of the zebra finch Z chromosome from the latest genome assembly (scaffold NC_045027 from genome version bTaeGut2.Pat.W.v2). The zebra finch scaffold has a long gap (consisting of N's) between the PAR and sex‐linked regions. The alignment was plotted using the AliTV v.1.0.6 browser tool (Ankenbrand et al., 2017)

ALLMAPS placed and arranged, that is, anchored, seven scaffolds (98a, 31, 5, 2, 134, 92 and 217) and the order remained the same when the PAR scaffold (217) was excluded (Supporting Information 3). We present the Z chromosome in an order where the PAR is at the beginning, followed by the ancestral‐Z and the added‐Z at the end, that is, 217(−) 92(+) 134(+) 2(+) 5(−) 31(+) 98a(−), as this follows the direction of previous avian Z chromosomes (Figure 1a). The seven anchored scaffolds correspond to 87.54 Mbp of the Z chromosome. ALLMAPS could not anchor scaffolds 94 (1.18 Mbp) and 4139 (0.03 Mbp), as they both had only 1 SNP. Based on their coverage, synteny information (to zebra finch, great tit and collared flycatcher Z) and almost complete lack of genetic variation, their likely position is in the NONREC region. Further confirmation that scaffold 217 (with nine assigned markers) represents the PAR of the great reed warbler Z chromosome, in addition to the pattern of linkage, comes from (i) the analysis of female/male coverage showing a pattern similar to autosomes and different to the rest of the Z chromosome (Figure 1b), and (ii) conserved synteny to the zebra finch scaffold “Z random” (Z‐linked but not ordered scaffolds) and the collared flycatcher's PAR contigs (Smeds et al., 2014). Alignment of the great reed warbler PAR and the first sex‐linked scaffold (scaffold 92) with the flycatcher PAR, and the first 1.2 Mbp of the new assembly‐version of the zebra finch Z chromosome (Rhie et al., 2020), is shown in Figure 1c.

### 3.2 Recombination rate

**FIGURE 2:**
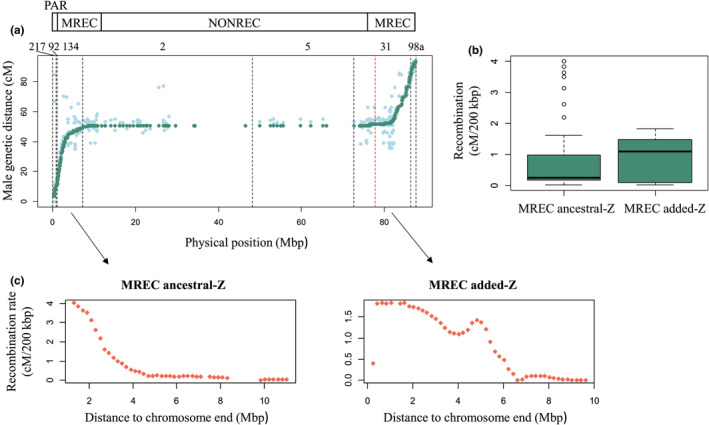
The recombination landscape of great reed warbler Z chromosome. (a) Male genetic distance as a function of the physical distance. Genetic marker order for the full Z chromosome, which was used to order the Z‐linked scaffolds, is shown in light green. Re‐evaluated genetic distances based on the final physical positions of markers are shown in dark green (evaluated only for the anchored Z scaffolds). All genetic map positions are based on male recombination. Ancestral part of the Z is in the beginning of the chromosome (starting with the PAR) and the added region is in the end, and the red dashed line shows the approximate border between the ancestral‐ and added‐Z (sizes 77.9 and 9.6 Mbp, respectively). Black dashed lines mark scaffold (217, 92, 134, 2, 5, 31 and 98a) borders. Above the plot is a schematic illustration of the three recombination regions of great reed warbler Z chromosome: PAR (where both sexes recombine), male‐recombining regions at both ends (where only males recombine, MREC) and nonrecombining region (no recombination in neither sex, NONREC). (b) Recombination rate within the MREC compared between ancestral‐ and added‐Z. (c) Relationship between recombination rate and distance to chromosome ends within the MREC, presented separately for the ancestral‐ and added‐Z

The re‐evaluation of genetic distances for the full Z gave a map length of 90.19 cM corresponding to a physical region of 87.54 Mbp, but with settings not fully suitable for the PAR. The sex‐linked Z (excluding the PAR) had a total map length of 83.69 cM corresponding to a physical region of 86.48 Mbp (Figure 2a). Dividing the genetic map length with the physical length gives an average recombination rate of 1.03 cM/Mbp including the PAR, and 0.97 cM/Mbp across the male‐specific Z (excluding the PAR). However, male recombination ceased completely between positions 8.47 and 76.42 Mbp (at genetic map position 50.90 cM; Figure 2a). Thus, a 67.94 Mbp region (i.e., 77.6% of the chromosome including the PAR) does not recombine in either sex in our pedigree data. This forms three regions to the recombination landscape of the great reed warbler Z chromosome: PAR (where both sexes recombine), MREC (male‐recombining Z) and NONREC (nonrecombining Z; Figure 2a). The average recombination rate was 4.52 cM/Mbp, when estimated for the MREC region only.

The MREC region is located in each end of the neo‐Z chromosome and can therefore be divided into an ancestral‐ and an added‐Z. The recombination rate was 5.41 cM/Mbp for the former and 3.90 cM/Mbp for the latter (i.e., 1.4 times higher in the ancestral‐Z). However, when the rates were compared between these regions using the 200 kbp data, they were not statistically different (Mann–Whitney U test: *U* = 1040.5, *p* = .66; Figure 2b).

The recombination rate depended strongly on physical position as it increased significantly towards the chromosome ends in both the ancestral‐ and added‐Z MREC parts (Spearman's correlation: *ρ* = −.99, *p* < 2.2e‐16, and *ρ* = −.92, *p* < 2.2e‐16, respectively). The relationship between recombination rate and chromosomal position showed an almost exponential shape in the ancestral‐Z, while for the added‐Z the pattern was less clear (Figure 2c).

### 3.3 Recombination rate in relation to genome characteristics

**FIGURE 3:**
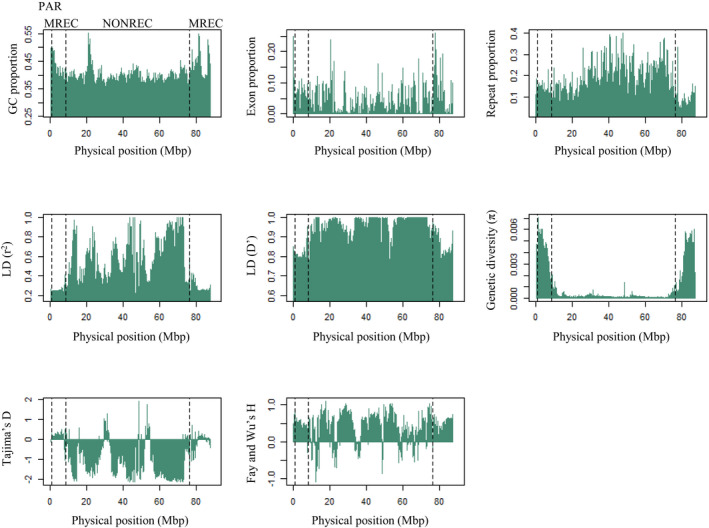
Genome characteristics, that is, GC content, exon proportion, repeat proportion, linkage disequilibrium (measured with r^2^ and D′), genetic diversity, Tajima's D and Fay and Wu's H, measured in 200 kbp bins along the great reed warbler Z chromosome. Dashed lines mark the boundaries between the three types of recombination regions: PAR (pseudoautosomal region, where both sexes recombine), MREC (male‐recombining region) and NONREC (nonrecombining region)

**FIGURE 4:**
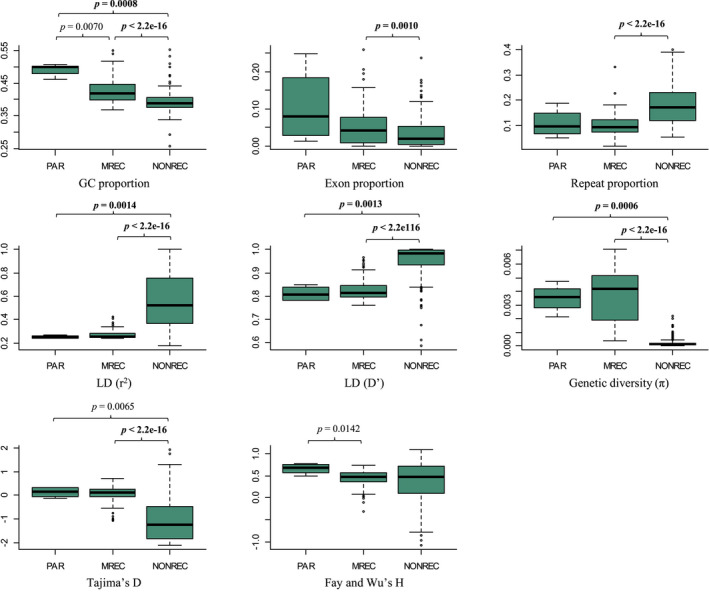
Comparison of genomic variables (GC content, exon proportion, repeat proportion, linkage disequilibrium measured with r^2^ and D′, genetic diversity, Tajima's D and Fay and Wu's H) between the three recombination regions (PAR, MREC and NONREC) of the great reed warbler Z chromosome. Definitions for the recombination regions are: PAR (pseudoautosomal region, where both sexes recombine), MREC (male‐recombining region) and NONREC (non‐recombining region). All data are evaluated in 200 kbp bins. *p*‐Values are shown for the statistically significant differences (Mann–Whitney U test, *p* < .05) between regions. *p*‐Values that remained significant after Holm's correction (1979) for multiple testing are marked in bold

Eight different genome characteristics were calculated in 200 kbp windows along the Z chromosome as shown in Figure 3. The relationships between these genome characteristics and recombination were first contrasted between the three recombination regions (PAR, MREC, NONREC; Figure 4). The GC content was significantly different between all three regions, showing the highest value in the PAR and the lowest in the NONREC region. Exon proportion was significantly higher in the MREC compared to the NONREC. The repeat proportion in turn was significantly lower in the MREC than in the NONREC. Linkage disequilibrium was significantly lower in the PAR and MREC when compared to the NONREC (for both r^2^ and D′). Genetic diversity and Tajima's D were in turn significantly higher in the PAR and MREC compared to the NONREC (Figure 4). Fay and Wu's H was significantly higher in the PAR compared to the MREC. Since the PAR had only four 200 kbp bins, its comparisons need to be interpreted cautiously due to the small sample size. Moreover, several of the tests involving the PAR (GC and Fay and Wu's H between MREC and PAR and Tajima's D between NONREC and PAR) were nonsignificant after correction the *p*‐value for multiple testing with Holm's corrections (*α* = .05, *m* = 24, i.e., 8 parameters and 3 regions, see Figure 4).

When the genome characteristics were compared between the ancestral‐ and added‐Z within the MREC, Tajima's D (Mann–Whitney U test: *U* = 552, *p* = .0003) and repeat proportion (*U* = 270, *p* = 2.8e‐10) showed a significant difference between these regions (both were higher in the ancestral‐Z). Also, LD measured with D′ showed significantly higher values in added‐Z (Mann–Whitney U test: *U* = 1338, *p* = .0037). The remaining parameters did not differ between the ancestral‐ and added‐Z parts of MREC (*p* > .05).

**FIGURE 5:**
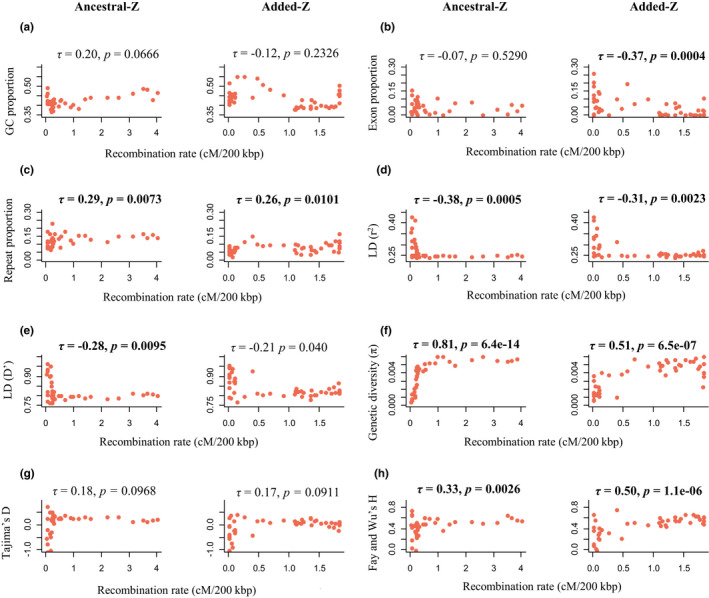
Correlations between recombination rate (cM/200 kbp) and genomic variables: (a) GC content, (b) exon proportion, (c) repeat proportion, (d) linkage disequilibrium (measured with r^2^), (e) linkage disequilibrium (measured with D′), (f) genetic diversity, (g) Tajima's D and (h) Fay and Wu's H on the great reed warbler Z chromosome. Correlations were assessed in 200 kbp bins, separately within the ancestral‐ and added‐Z. correlations were tested with Kendall's *τ* and *p*‐values that remained significant after Holm's correction (1979) for multiple testing are marked in bold

Next, we evaluated the relationship between recombination rate and genome characteristics within the MREC. Genetic diversity (Kendall rank correlation: *τ* = 0.63, *p* < 2.2e‐16), repeat proportion (*τ* = 0.20, *p* = .0054) and Fay and Wu's H (*τ* = 0.42 *p* = 5.4e‐09) correlated positively, while exon proportion (*τ* = −0.25, *p* = .0005) and linkage disequilibrium (r^2^; *τ* = −0.33, *p* = 6.1e‐06 and D′; *τ* = −0.21, *p* = .0037) correlated negatively, with the recombination rate. The remaining correlations were nonsignificant. When these associations were tested separately for the ancestral‐ and added‐Z (Figure 5a–h), linkage disequilibrium and genetic diversity retained strong negative and strong positive correlations, respectively, in both regions (Figure 5d–f). However, the negative correlation between exon proportion and recombination was found only in the added‐Z part of MREC (Figure 5b). Recombination rate had positive association with proportion of repeats as well as Fay and Wu's H in both regions (Figure 5c–h). Most of the significant associations remained significant (all within ancestral‐Z and 7 of 8 within added‐Z) after correction the *p*‐value for multiple testing with Holm's corrections (*α* = .05, *m* = 8, i.e., 8 parameters tested separately for both two regions, see Figure 5a–h).

Due to the somewhat unexpected observation of a positive correlation between recombination rate and repeat proportion, we further analysed the major types of repeats separately: DNA transposons, LINEs, SINEs, LTRs, unknown repeats, low complexity repeats and simple repeats (see Figure S3 and Supporting Information 5 for results and discussion).

### 3.4 Gene‐specific evolutionary rates

**FIGURE 6:**
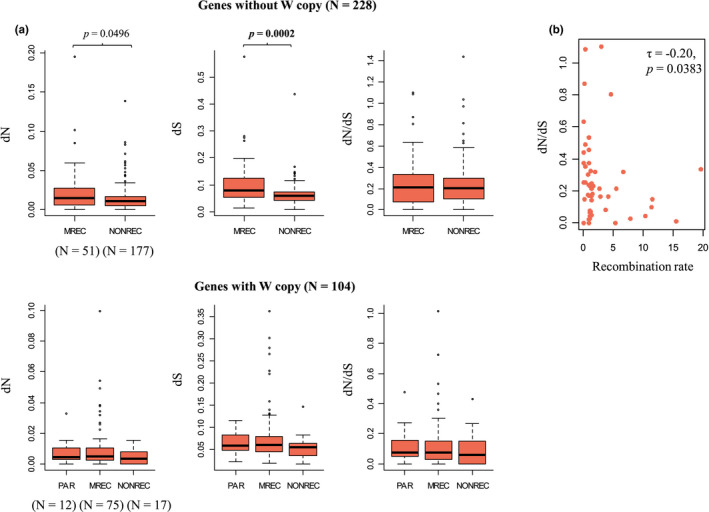
Branch‐specific evolutionary rates (dN, dS and dN/dS) calculated for the great reed warbler Z‐linked and PAR genes. Branch‐specific rates were calculated using orthologous genes from zebra finch and chicken. Rates were calculated separately for genes that have retained their W copy (PAR genes and Z‐linked genes) and ones that have lost W copy. (a) Rates were compared pairwise between three recombination regions (PAR, MREC and NONREC) of the great reed warbler Z chromosome. Definitions for the recombination regions are: PAR (pseudoautosomal region, where both sexes recombine), MREC (male‐recombining region) and NONREC (nonrecombining region). Statistically significant (Mann–Whitney U test *p* < .05) rate differences between regions are shown and *p*‐values that remained significant after Holm's correction (1979) for multiple testing are marked in bold. (b) Evolutionary rates were correlated with recombination rate and the only significant correlation was observed with dN/dS and recombination in genes without W copy (nonsignificant correlations not shown). However, this result did not remain significant after Holm's correction (1979) for multiple testing

Whether a Z chromosome gene has lost its gametologous W copy or not is strongly associated with its evolutionary rates (dN, dS and dN/dS; Sigeman et al., 2021), and we took this into account by analysing genes with and without W copy separately. For genes with W copy, none of the rates (dN, dS and dN/dS) differed significantly among the PAR, MREC and NONREC (Figure 6). However, for genes without W copy, dS and dN were significantly higher in the MREC compared to the NONREC (Mann–Whitney U test: *U* = 6067, *p* = .0002 and *U* = 5329, *p* = .0496, respectively; Figure 6).

Next, we ran the same comparisons, but separating the MREC into ancestral‐ and added‐Z. For genes with W copy, dN was significantly higher in the PAR and added‐Z compared to the ancestral‐Z (Mann–Whitney U test: *U* = 10, *p* = .0396 and *U* = 74.5, *p* = .0337, respectively). Further, dN/dS was higher in the added‐Z compared to the ancestral‐Z (Mann–Whitney U test: *U* = 81, *p* = .0467). However, MREC ancestral‐Z had only five genes with remaining W copy, so these results need to be interpreted cautiously. Moreover, after correction the *p*‐value for multiple testing with Holm's corrections (*α* = .05, *m* = 24, i.e., 3 rates and all region comparisons included), none of the region comparisons remained statistically significant for genes with W copy (Figure 6). For genes without W copy, dS was significantly higher in the MREC ancestral‐Z compared to the NONREC (*U* = 4791, *p* = 4.1e‐05). After correction the *p*‐value f or multiple testing with Holm's corrections (*α* = .05, *m* = 12, i.e., 3 rates and all region comparisons included), only the results for dS remained statistically significant for genes without W copy (Figure 6).

When we tested the gene‐specific correlations between the amount of recombination and evolutionary rates (within MREC), none of the correlations were significant for genes with W copy, whereas in genes without W copy the rate correlated negatively with dN/dS in MREC (Kendall rank correlation: *τ* = −0.20, *p* = .0383; Figure 6.) and also separately in ancestral‐Z (*τ* = −0.24, *p* = .0369) and positively with dS in ancestral‐Z (*τ* = 0.24, *p* = .0346). After correction the *p*‐value for multiple testing with Holm's corrections (*α* = .05, *m* = 9, i.e., 3 rates and all correlations included), all correlations became statistically nonsignificant also for genes without W copy.

## 4 DISCUSSION

### 4.1 Recombination landscape along the great reed warbler Z chromosome

Based on our pedigree data, most of the great reed warbler Z chromosome (77.6%) is not recombining at all in either of the sexes (the female‐specific W lacks recombination outside the PAR). This creates three distinct regions within the chromosome: the small PAR where both sexes recombine, the male‐recombining Z (MREC) and the nonrecombining Z (NONREC). The nonrecombining region is probably slightly larger (up to 79%) as the two (small) un‐anchored sex‐linked scaffolds (94 and 4139) most probably originate from this region. In a typical case of heteromorphic sex chromosomes, the chromosome pair does not recombine in the heterogametic sex (Charlesworth et al., 2005), but in the great reed warbler recombination along a large section of the Z chromosome is also absent (or exceedingly rare) in the homogametic sex.

Centromeric regions often have lower recombination rates compared to chromosome ends and this bias in recombination towards telomeres is found especially in vertebrate males, while females often show more evenly distributed patterns (Sardell & Kirkpatrick, 2020). Our preliminary results from a genome‐wide linkage map of the great reed warbler suggest that the males have telomere‐biased recombination also at autosomes while females recombine more evenly along the chromosome length (Ponnikas et al., 2022). Thus, the observed recombination landscape of the Z chromosome does not seem to be the result of the sex chromosome fusion per se, although the neo‐sex chromosome formation made an expansion of the nonrecombining region (NONREC) possible by increasing the total length of the sex chromosomes. In XY‐systems, telomere‐biased recombination in males (XY) can facilitate the evolution of sex chromosomes as genomic regions with low rates of recombination may be generally predisposed to evolve sex‐linked regions (Sardell & Kirkpatrick, 2020) and this has been suggested to occur in both guppies (Bergero et al., 2019) and in *Rumex* plants (Rifkin et al., 2021). This situation can further lead to a high rate of sex chromosome turnover, as suggested in frogs where males only recombine at the ends of chromosomes (Jeffries et al., 2018). In such study systems, sudden linkage of a very high number of genes to the Y chromosome is expected to generate particularly strong Hill–Robertson interferences and induce rapid accumulation of deleterious‐mutation load along the entire Y chromosome (except at the recombining tips); a situation that can quickly select for turnovers. In contrast to the male heterogametic systems, telomere‐biased male recombination in ZW‐systems has different effects on sex chromosome evolution and leads to a situation where potentially large regions of the sex chromosomes do not recombine in either of the sexes.

Our pedigree represents only a proportion of the total amount of meioses and potential crossing over events in the population, so we cannot exclude the possibility of rare recombination events within the NONREC region. However, the drastic loss of diversity and accumulation of repeats within the central parts of the Z chromosome are in line with a scenario where different processes (both drift and selection) have acted on a region with no or very low amount of recombination over a long evolutionary time scale, and thus that restricted recombination in the large central part of the Z chromosome has been a consistent feature over a relatively long period of time in the great reed warbler. The crossing over events cluster at the chromosome ends, where the recombination rate showed further fine‐scale dependence on the chromosomal position by increasing towards the ends. This pattern is in line with the general bias of recombination towards the telomeres in male vertebrates (Sardell & Kirkpatrick, 2020). When compared to other bird species, the zebra finch shows a similar strong telomeric effect in its recombination rates and has a comparable recombination desert in the central parts of its Z chromosome (Backström et al., 2010). A less pronounced telomeric effect (without the centromeric recombination deserts) have been observed also in chicken (Groenen et al., 2009) and in the collared flycatcher (Kawakami et al., 2014). It is generally thought that each chromosome arm requires at least one chiasma (resulting in a minimum of 50 cM distance) for efficient chromosome pairing and segregation during meiosis (Page & Hawley, 2003). As our results show a shorter total map distance (87.54 cM including two arms), we still miss some of the recombination events in the Z chromosome, especially as the genetic map for the PAR could not be resolved with the current data. The previous linkage maps for the study species have found Z chromosome map lengths of 45.3 cM (Hansson et al., 2005), 155 cM (Åkesson et al., 2007) and 156.1 cM (Pala et al., 2012). All these maps were based on relatively few markers and missed telomeric recombination events. Moreover, the last two were partly based on AFLP markers, which are difficult to score and filter for genotyping errors as they are dominant and the recombination distances were probably inflated due to genotyping errors.

### 4.2 Great reed warbler PAR

**FIGURE 7:**
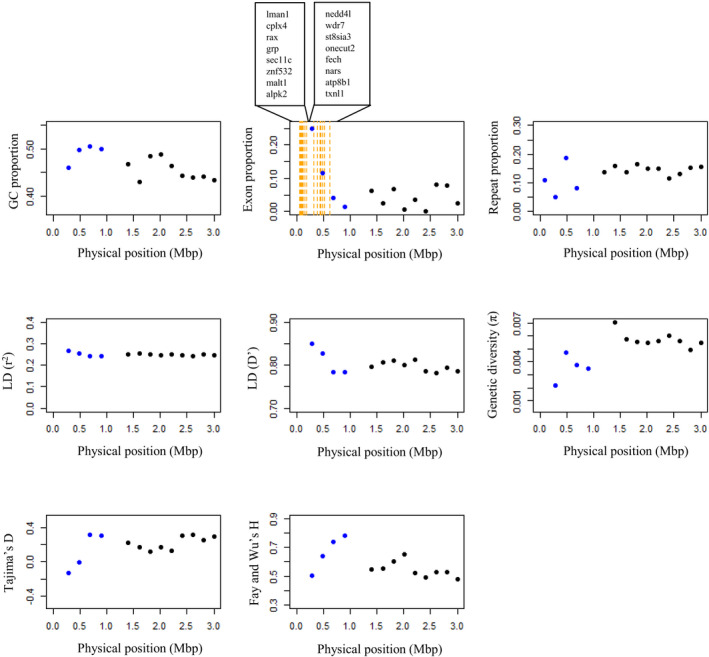
Genome characteristics, that is, GC content, exon proportion, repeat proportion, linkage disequilibrium (measured with r^2^ and D′), genetic diversity, Tajima's D and Fay and Wu's H, measured in the 200 kbp bins along the great reed warbler PAR (scaffold 217, marked with blue). For comparison, also shown is the first 10 bins in the sex‐linked Z (scaffold 92, marked with black). The graph describing the exon proportions also shows the positions for the 16 protein‐coding genes (in orange) in the PAR, which have been listed corresponding to their physical order in the sequence

The PAR has recently been described from several avian species (Smeds et al., 2014; Xu et al., 2019; Zhou et al., 2014). Of these species, the collared flycatcher arguably has the best description for a passerine PAR and our study adds another fine‐scale described PAR. The great reed warbler PAR covers approximately 1% of the total Z chromosome and it seems to be slightly larger (892 kbp) compared to collared flycatcher (630 kbp), which however was suspected to be incomplete (Smeds et al., 2014). After carefully checking the support for each of the PAR annotations, we could confirm 16 protein‐coding genes; the same found in the PAR of collared flycatcher (Figure 7; Smeds et al., 2014). Alignments to collared flycatcher and zebra finch showed two inversions in the PAR of our study species (Figure 1c).

The fusion between a part of chromosome 4A and the ancestral sex chromosomes in the great reed warbler and other Sylvioidea species (Sigeman et al., 2020) occurred to the sex‐linked end of the Z chromosomes and, based on our linkage map results, it does not seem to have created a second PAR as autosomally segregating markers were not assigned to this added‐Z end of the chromosome.

### 4.3 Recombination rate in relation to genome characteristics

The apparent lack of recombination in the central part of the Z chromosome shapes the evolution of this region drastically compared to the recombining parts, which is manifested as higher linkage disequilibrium and amount of repeats, as well as a lower GC content. This region also had a lower proportion of exonic sequence, but concluding that this is due to gene degeneration should be done cautiously as the higher amount of repeats in the NONREC could partly decrease the proportion of exons in the bins of a certain size. Indeed, exon density decreased with higher repeat density in NONREC (Kendall's rank correlation: *τ* = −0.24, *p* = 3.9e‐11) while in MREC there was no such connection (*τ* = −0.12, *p* = .0872). Further, centromeric regions (which make up part of the NONREC) tend to have lower gene density in general. Perhaps the clearest consequence from the apparent lack of recombination is the loss of genetic diversity. The level of genetic variation is determined by mutations, recombination, genetic drift, selection, GC‐biased gene conversion and demography (Sayres, 2018). The neutral expectation of sex‐linked Z chromosome diversity corresponds to 0.75 of the general autosomal level, which reflects the difference in the effective population sizes of these genomic regions (Z chromosome *N*_E_ is three quarters of the autosomal *N*_E_, assuming equal sex‐ratios). The fact that males have a higher mutation rate compared to females (male mutation bias; Kirkpatrick & Hall, 2004) can increase this Z/A neutral expectation above 0.75, as the Z chromosome spends ⅔ of its evolutionary time in males. On the other hand, higher variance in reproductive success in males may decrease this relative diversity below 0.75, through a decrease in male effective population size (*N*_E_‐males < *N*_E_‐females). Assuming that the PAR reflects roughly the general autosomal diversity level, the male‐specific Z (comprising the MREC and NONREC) shows a relative diversity of 0.27 compared to the PAR. If purely neutral, a ratio this low would need an unrealistically low *N*_E_‐males/*N*_E_‐females; too low to be explained by the polygynous mating system (Hasselquist, 1998) and a moderate male‐biased reproductive variance in great reed warblers (Hansson et al., 2004; Tarka et al., 2015). When the relative Z/A diversity was calculated separately for the MREC and NONREC regions, the former showed equal amounts of variation compared to autosomes (1.04; the slightly higher diversity of MREC could be due to the higher recombination rate per bp compared to the autosomes), while the latter had drastically lower value (0.06). The similar level of variation between the PAR and MREC (despite having different *N*_E_s) could result from male‐mutation bias in the latter. The difference in relative diversity values between the MREC and NONREC results most likely from the observed lack of recombination and following selection acting on several linked sites (linked selection) in the latter, since male mutation bias, effective population size, drift and demography should affect these two regions similarly.

The negative Tajima's D values in the NONREC supports the idea of linked selection causing a loss of polymorphism. Tajima's D compares two genetic diversity estimates, nucleotide diversity and segregating sites, and values below zero follow from excess of low frequency polymorphisms. The sex‐linked Z is mostly hemizygous in females due to degeneration of the W chromosome (within the NONREC 87.8% of the genes we used in the evolutionary rate analysis have lost its W copy), which enhances the effect of selection further, since also recessive alleles are exposed to selection in females. Fay and Wu's H was mostly positive or close to zero within the NONREC (median value 0.48), which is not expected if the reduction in diversity had been primarily caused by recent positive selection. Thus, we hypothesize that purifying selection has played an important role in lowering the diversity and causing negative Tajima's D values in this part of the neo‐sex chromosome. However, even in very detailed studies, separating the contribution of different selective processes on linked sites remains challenging (Stephan, 2010).

There were three intervals within the NONREC that showed high positive Tajima's D values and thus stood out from the general pattern of negative values in this region (Figure 3). Closer assessment of two of the wider intervals (positions ~29–32 Mbp and ~ 52–55 Mbp) suggested that the high Tajima's D values could have been caused by divergent haplotypes that are present at high frequency in the study population, perhaps due to introgression from another population or long‐term balancing selection. The low recombination rate in the region could contribute to maintain such quite long chromosomal chunks over time. The third, rather narrow interval (position ~48 Mpb), however, is more likely caused by erroneously collapsed paralogous sequences as it clearly shows a higher read coverage.

Linkage disequilibrium is strongly affected by recombination, which breaks the linkage between loci. Our results were in line with this, with LD being significantly higher in the NONREC and showing strong negative correlation with recombination rate. The positive association between recombination and GC content in the region comparisons was expected too, since crossover events are known to affect the nucleotide composition of sequences through GC‐biased gene conversion (Mugal, Arndt, et al., 2013) and since higher GC content is linked to recombination hotspots in many species across eukaryotes (Stapley et al., 2017a). In addition to the significantly lower genetic diversity in the NONREC, diversity increased strongly with the amount of recombination within the MREC. A positive correlation between genetic diversity and recombination is widely observed (Frankham, 2012) and both selection and neutral mechanistic processes can explain this association. In nonrecombining regions, like in the great reed warbler NONREC, linked selection can explain the loss of variation (Charlesworth, 2009; Mugal, Nabholz, et al., 2013), whereas in recombining regions the positive association could be additionally explained by the crossing over‐associated higher mutation rate (i.e., a neutral process). This is thought to result from the fact that repairing of the DNA double‐strand breaks during the crossing over event is mutagenic (Arbeithuber et al., 2015; Kulathinal et al., 2008).

As expected, the exon proportion was lower and the repeat proportion higher in the NONREC. A positive correlation is often found between gene density and recombination, which can be beneficial as recombination reduces the Hill‐Robertson interference between loci (Hill & Robertson, 1966). In contrast, the correlation between gene density and recombination within added‐Z was negative, which could suggest a selective advantage of linkage (see below). The higher repeat density in the NONREC most likely results from the decreased efficiency of selection to remove these sequences in the absence of recombination. However, correlations within added‐ and ancestral‐Z showed an opposite pattern (positive correlation), which we discuss below.

### 4.4 Comparing the ancestral‐ and added‐Z

Crossover events seem to have happen approximately equally often in the ancestral‐ and added‐Z within the MREC. However, the recombination rate correlations with distance to chromosome end had slightly different patterns between these regions (Figure 2c). The ancestral‐Z showed clear dependence between the rate and the physical position, suggesting that the rate variation is not selectively driven by the gene content. The pattern along the added‐Z in turn deviated somewhat from an exponential association between physical position and recombination rate. This could have been caused by many factors. For example, a lack of informative SNPs in the bins where the rate drops from the expected could be one simple explanation. Selection could be another reason for this deviation, if the fitness effect of gene linkage varies along the added‐Z. Then, selection could create a local rate that is higher or lower than expected based on the physical position. This possible difference in selective regimes between ancestral‐ and added‐Z was supported by significantly lower Tajima's D in the added‐Z. While the Tajima's D values were mostly positive in the ancestral‐Z, half of the added‐Z region showed negative values (Figure 3), suggesting linked selection. Linkage disequilibrium (measured with D′) was also significantly higher in the added‐Z. Selectively advantageous gene linkage in added‐Z is further supported by the significant negative correlation between gene density and recombination rate. Further studies are needed to assess this possible variation in the fitness effect of gene linkage along the Z chromosome.

In addition to the possible selective differences (due to gene content) between ancestral‐ and added‐Z, the age difference in the sex‐linkage could have affected the sequence patterns between these regions. Some of the unexpected correlation patterns for the added‐Z, such as the lack of correlation between recombination and GC content, could be caused by the fusion history of this region. As the recombination rate depends on the physical position along the chromosome, the current recombination landscape of the added‐Z could differ from the previous autosomal one and this change could lead to the situation where the GC content reflects both previous autosomal and current recombination rate effects. This could disrupt the sign of ongoing GC biased gene conversion and the following expected positive correlation with recombination rate. Ancestral‐Z had higher repeat proportion, while both MREC regions showed positive correlation between recombination rate and repeats. Thus, the amount of repeats seems to be a complex result of age of sex‐linkage and varying sign of correlation between recombination rate and different repeat types (Figure S3 and Supporting Information 5).

### 4.5 Gene‐specific evolutionary rates

Even though the apparent lack of recombination and the following linked selection has decreased the genetic diversity in the NONREC, the efficiency of selection at protein‐coding sequence does not seem to have been hampered, since dN and dN/dS had similar values between the NONREC and MREC. The lack of association was further strengthened by the nonsignificant correlations between dN, dN/dS and recombination rate. A recent study on white‐eyes (*Zosterops* spp.), another Sylvioidea genus that shares the same neo‐sex chromosome system as the great reed warbler, also found that added‐Z genes and autosomal genes had similar dN/dS values despite the lower recombination rate on chromosome Z (as inferred from low GC content, Leroy et al., 2021). Further, a neo‐sex chromosome in monarch butterfly (*Danaus plexippus*) showed evolutionary rate variation depending on both the age of sex‐linkage (added‐ and ancestral‐Z regions) and the gene expression pattern, highlighting the role of other factors in addition to recombination affecting these rates (Mongue et al., 2021).

The significantly higher dS in the MREC compared to the NONREC in genes without W copy could be caused by mutagenic effects of recombination and/or GC‐biased gene conversion associated with recombination. It could have been expected that the NONREC would show higher dN/dS rate as a result of relaxed purifying selection (i.e., there is a negative association between recombination and evolutionary rate), but instead our results show nonsignificant association. A similar kind of lack of association has been observed for example in primates (Bullaughey et al., 2008). However, previous studies in birds have found significant associations between recombination and evolutionary rates (Gossmann et al., 2014), and in addition to Hill‐Robertson interferences, GC‐biased gene conversion was shown to be the mechanism explaining the association (Bolívar et al., 2016; Rousselle et al., 2019). The lack of associations in our analyses could have many reasons. Rare recombination events within the NONREC region that our pedigree data did not record could be enough to enable selection to work efficiently. Since the level of genetic diversity is very low in the NONREC region, recombination events could occur unnoticed too as crossover might not lead to allelic change (this is, however, unlikely as the drop in recombination occurs already within the MREC, which strongly supports that recombination occurs predominantly in the chromosome ends). Second, the sample sizes were rather low for many of the comparisons within diploid and hemizygous genes (i.e., with and without W copy; Figure 6), which could have lowered the statistical power of our analyses. Broadening the analyses across the whole genome, and assessing the roles of GC‐biased gene conversion and gene expression patterns, could be future aims to further understand the associations between recombination rate variation and evolutionary rates.

## 5 CONCLUSIONS

The great reed warbler Z chromosome shows an extreme variation in male recombination rate and our results confirm a role of linked selection in explaining the positive association between crossover rate and overall diversity in the genome. Nonetheless, we did not observe reduced efficiency of selection on protein‐coding sequences despite the apparent lack (or very low level) of recombination. Possibly, rare recombination within the NONREC may happen often enough to prevent accumulation of slightly deleterious mutations. The fused added‐Z region seems to reflect previous autosomal and current Z‐linked recombination landscapes in its sequence characteristics. This region showed slightly different recombination landscape compared to the ancestral‐Z, and further research is needed to test if this is due to its gene content and the following differences in selective regimes.

## AUTHOR CONTRIBUTIONS

Suvi Ponnikas and Bengt Hansson designed the study; Suvi Ponnikas, Max Lundberg and Hanna Sigeman performed analyses, and Suvi Ponnikas wrote the manuscript with input from all coauthors.

## CONFLICTS OF INTERESTS

The authors declare that they have no conflict of interest.

## Supporting information


Supinfo

## Data Availability

The RADseq data used in this study are available under NCBI BioProject PRJNA445660. The great reed warbler reference genome (Acrocephalus arundinaceus, GenBank accession GCA_021534815.1) is available under NCBI BioProject PRJNA765537. Whole‐genome re‐sequencing data from the five great reed warbler males (SRR16079849, SRR16079850, SRR16079851, SRR16079861 and SRR16079862) and the male clamorous reed warbler (Acrocephalus stentoreus, SRR16079857) are accessible under NCBI BioProject PRJNA578893.
