# Supplementary material for: Extreme variation in recombination rate and genetic diversity along the Sylvioidea neo‐sex chromosome
Source: Mol Ecol. 2022 Jun 6;31(13):3566–83. doi: 10.1111/mec.16532 (PMC9327509; doi:10.1111/mec.16532)
Supplement: Supplementary file 1 — Supinfo [file MEC-31-3566-s001.docx]

**Supplemental Information for:**

**Extreme variation in recombination rate and genetic diversity along the Sylvioidea neo-sex chromosome**

Ponnikas, S., Sigeman, H., Lundberg, M. & Hansson, B.

**Table of Contents:**

| **Supplementary Material 1** | Page 2 |
| --- | --- |
| **Supplementary Figure 1** | Page 3 |
| **Supplementary Material 2** | Page 4 |
| **Supplementary Figure 2** | Page 10 |
| **Supplementary Material 3** | Page 11 |
| **Supplementary Figure 3** | Page 12 |
| **Supplementary Material 4** | Page 14 |
| **Supplementary Material 5** | Page 16 |

**Supplementary Material 1**.

Description of the three pipelines used for variant calling and filtering of RAD-seq data. If not stated otherwise, all filtering steps in different pipelines were performed in VCFtools v. 0.1.14 (Danecek et al. 2011).

In the first pipeline, variant calling was done with freebayes v1.1.0 (Garrison & Marth 2012) and the raw variants output contained 2.55 million SNPs in 3123 scaffolds. Filtering was performed following the SNP filtering approach in dDocent v. 2.2.20 (Puritz et al. 2014), when applicable to our data. We did not include the HWE-filtering step (as our data consists of relatives) or the rad_haplotyper script. Then, we applied two additional filtering steps, which kept only bi-allelic sites and removed sites overlapping with annotated repeats. Repetitive regions were identified by (i) using a bed-file of repeats constructed using a repeat library (fAlb15_rm3.0_aves_hc.lib; provided by A. Suh, Uppsala University) containing Repbase repeats (Bao et al. 2015) from chicken (*Gallus gallus*; Hillier et al. 2004) and zebra finch (*Taeniopygia guttata*; Warren et al. 2010), curated hooded crow (*Corvus corone*) repeats (Vijay et al. 2016), and curated collared flycatcher (*Ficedula albicollis*) repeats (Suh et al. 2018), and (ii) *de novo* prediction by RepeatModeler (raw output, no manual curation). After filtering, 66 392 SNPs in 344 scaffolds remained. Lastly, four individuals were removed after this filtering step because of too much missing data (> 85 %), which left 263 birds in the data set.

In the second pipeline, SNPs were called with freebayes as above, but filtering was different. We removed any indels, which was followed by removal of sites with quality value below 30, removal of genotypes with less than 8x coverage and with more than twice the average autosomal coverage. Then, we removed SNPs with average minDP<15 and average maxDP>200 and sites with more than 50% of missing data. Lastly, we removed sites with minimum allele frequency below 0.05, SNPs overlapping repeats and kept only biallelic sites. This pipeline resulted in 137 507 SNPs in 622 scaffolds.

In the third pipeline, SNP calling was done with mpileup in samtools v. 1.4 (settings ‐t DP and ‐t SP to keep per‐sample read depth and strand bias, and flags ‐A to keep anomalous read pairs in variant calling and -g to compute genotype likelihoods and output them in the binary call format) and the call command in bcftools v. 1.6 (with flags -vm to use multiallelic calling model and to output variant sites only). The raw output before filtering consisted of 3.97 million variants. We filtered the data mainly following Hansson et al. (2018), with two additional steps including removal of sites overlapping repeats (see above) and with quality score < 30. This resulted in 209 126 SNPs in 712 scaffolds.

The final set of SNPs was extracted from the output of the first pipeline (called with freebayes and filtered with dDocent) using bcftools isec by selecting SNPs that were shared by all three pipeline outputs (position and exact allele match required). This data set had 50 614 SNPs in 328 scaffolds. As the female-specific W chromosome cannot be ordered using linkage mapping (as it does not recombine), we removed known W-linked contigs (see Sigeman et al. 2020b) from the data. As the data had been mapped to an earlier version of the reference genome (Sigeman et al. 2020b), we removed all SNPs located on scaffolds identified as redundant in the final assembly.


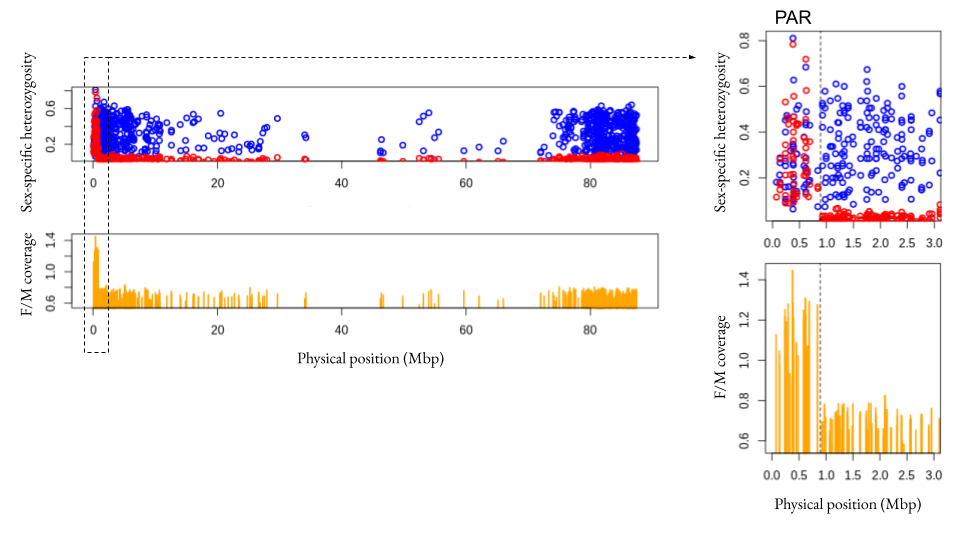


**Supplementary Figure 1**. Sex-specific heterozygosity (blue: males; red: females) and female/male coverage ratio (yellow) based on RAD-seq SNP data along the great reed warbler Z chromosome. Here, the values have been calculated along Z-linked scaffolds that were anchored (ordered and oriented) in ALLMAPS analyses. We have included all filtered markers from these scaffolds (*N* = 906), i.e., including also those that were not individually assigned to the Z linkage group, to get best possible coverage across the chromosome. The zoomed in PAR region in the beginning (0-892 kbp; border marked with dashed line) shows an autosomal patterns for both coverage and heterozygosity, while the rest of the chromosome shows clear sex-linked patterns: i) females show approximately half of the mapping depth to the Z compared to males, and ii) females are (close to) homozygous (since they are hemizygous) for Z-linked loci. Both of these patterns are caused by divergence or loss of the W-copy in females.

**Supplementary Material 2.** Scripts to produce the linkage map and recombination rate estimates:

********************************************************

########################################################

# LINKAGE MAPPING IN Lep-MAP3

########################################################

********************************************************

******************************

# Lep-MAP3: ParentCAll2

******************************

# In this step full genome data.

java -cp bin/ ParentCall2 vcfFile=filtered_final_file.vcf ZLimit=2 removeNonInformative=1 halfSibs=1 data=Pedigree.txt > DATA_CALL_file

********************************

# Lep-MAP3: Filtering2

********************************

# In this step full genome data.

java -cp bin/ Filtering2 dataTolerance=0.01 removeNonInformative=1 data=DATA_CALL_file > DATA_CALL_file_FIL_1

***************************************

# Lep-MAP3: SeparateChromosomes2

***************************************

# In this step full genome data.

java -cp bin/ SeparateChromosomes2 lodLimit=16 sizeLimit=23 data=DATA_CALL_file_FIL_1 numThreads=11 > DATA_CALL_file_FIL_1_MAP.txt

# Here, Z chr was combined into one LG (LG10, see manuscript for details).

*************************************

# Lep-MAP3: JoinSingles2All.

*************************************

# In this step full genome data.

#[LOD 15; lodDiff=5]

java -cp bin/ JoinSingles2All lodLimit=15 lodDifference=5 map=DATA_CALL_file_FIL_1_MAP.txt data=DATA_CALL_file_FIL_1 numThreads=11 > LOD15_5.txt

#[LOD 14; lodDiff=5]

java -cp bin/ JoinSingles2All lodLimit=14 lodDifference=5 map=./LOD15_5.txt data=DATA_CALL_file_FIL_1 numThreads=11 > ./LOD14_5.txt

#[LOD 13; lodDiff=5]

java -cp bin/ JoinSingles2All lodLimit=13 lodDifference=5 map=./LOD14_5.txt data=DATA_CALL_file_FIL_1 numThreads=11 > ./LOD13_5.txt

#[LOD 12; lodDiff=5]

java -cp bin/ JoinSingles2All lodLimit=12 lodDifference=5 map=./LOD13_5.txt data=DATA_CALL_file_FIL_1 numThreads=11 > ./LOD12_5.txt

#[LOD 11; lodDiff=5]

java -cp bin/ JoinSingles2All lodLimit=11 lodDifference=5 map=./LOD12_5.txt data=DATA_CALL_file_FIL_1 numThreads=6 > ./LOD11_5.txt

#[LOD 10; lodDiff=5]

java -cp bin/ JoinSingles2All lodLimit=10 lodDifference=5 map=./LOD11_5.txt data=DATA_CALL_file_FIL_1 numThreads=6 > ./LOD10_5.txt

#[LOD 9; lodDiff=5]

java -cp bin/ JoinSingles2All lodLimit=9 lodDifference=5 map=./LOD10_5.txt data=DATA_CALL_file_FIL_1 numThreads=6 > ./LOD9_5.txt

#[LOD 8; lodDiff=5]

java -cp bin/ JoinSingles2All lodLimit=8 lodDifference=5 map=./LOD9_5.txt data=DATA_CALL_file_FIL_1 numThreads=6 > ./LOD8_5.txt

#[LOD 7; lodDiff=5]

java -cp bin/ JoinSingles2All lodLimit=7 lodDifference=5 map=./LOD8_5.txt data=DATA_CALL_file_FIL_1 numThreads=6 > ./LOD7_5.txt

#[LOD 6; lodDiff=4]

java -cp bin/ JoinSingles2All lodLimit=6 lodDifference=4 map=./LOD7_5.txt data=DATA_CALL_file_FIL_1 numThreads=6 > ./LOD6_4.txt

#[LOD 5; lodDiff=4]

java -cp bin/ JoinSingles2All lodLimit=5 lodDifference=4 map=./LOD6_4.txt data=DATA_CALL_file_FIL_1 numThreads=6 > ./LOD5_4.txt

#[LOD 4; lodDiff=4]

java -cp bin/ JoinSingles2All lodLimit=4 lodDifference=4 map=./LOD5_4.txt data=DATA_CALL_file_FIL_1 numThreads=6 > ./LOD4_4.txt

#[LOD 3; lodDiff=4]

java -cp bin/ JoinSingles2All lodLimit=3 lodDifference=4 map=./LOD4_4.txt data=DATA_CALL_file_FIL_1 numThreads=6 > ./LOD3_4.txt

#[LOD 3; lodDiff=3]

java -cp bin/ JoinSingles2All lodLimit=3 lodDifference=3 map=./LOD3_4.txt data=DATA_CALL_file_FIL_1 numThreads=6 > ./LOD3_3.txt

#[LOD 3; lodDiff=2; ran twice]

java -cp bin/ JoinSingles2All lodLimit=3 lodDifference=2 map=./LOD3_3.txt data=DATA_CALL_file_FIL_1 numThreads=6 > ./LOD3_2.txt

java -cp bin/ JoinSingles2All lodLimit=3 lodDifference=2 map=./LOD3_2.txt data=DATA_CALL_file_FIL_1 numThreads=6 > ./LOD3_2B.txt

**************************************************

# Lep-MAP3: OrderMarkers2 (within the LGs).

**************************************************

# Ordering was originally done for all linkage groups simultaneously. Here scripts only for Z chr (LG 10). Run 10 times. Order with and without PAR markers.

value="10"

for value in ${value}; do java -Xmx20g -cp bin/ OrderMarkers2 map=DATA_CALL_file_FIL_1_MAP.txt data=DATA_CALL_file_FIL_1 chromosome=${value} numThreads=11 grandparentPhase=1 minError=0.001 useKosambi=1 recombination2=0 > ./Order_LG${value}_1.txt; done

for value in ${value}; do java -Xmx20g -cp bin/ OrderMarkers2 map=DATA_CALL_file_FIL_1_MAP.txt data=DATA_CALL_file_FIL_1 chromosome=${value} numThreads=11 grandparentPhase=1 minError=0.001 useKosambi=1 recombination2=0 > ./Order_LG${value}_2.txt; done

for value in ${value}; do java -Xmx20g -cp bin/ OrderMarkers2 map=DATA_CALL_file_FIL_1_MAP.txt data=DATA_CALL_file_FIL_1 chromosome=${value} numThreads=11 grandparentPhase=1 minError=0.001 useKosambi=1 recombination2=0 > ./Order_LG${value}_3.txt; done

...

for value in ${value}; do java -Xmx20g -cp bin/ OrderMarkers2 map=DATA_CALL_file_FIL_1_MAP.txt data=DATA_CALL_file_FIL_1 chromosome=${value} numThreads=11 grandparentPhase=1 minError=0.001 useKosambi=1 recombination2=0 > ./Order_LG${value}_10.txt; done

# Get likelihoods for all LGs in the ten runs.

grep "likelihood" Order_LG* > AllLikelihoods.txt

# Open in Excel, sort first by LG (ASCENDING) and then by Likelihood (DESCENDING). This way the best order for each LG is always first. Save the file.

cat AllLikelihoods.txt | sed -n "1~5p" | cut -f 1 | tr -d ':#***' > BestLikelihoods.txt

# Calculate distances for best order (use only Male dist.)

java -Xmx20g -cp bin/ OrderMarkers2 evaluateOrder=./Order_LG10_xx.txt improveOrder=0 data=DATA_CALL_file_FIL_1 numThreads=11 grandparentPhase=1 minError=0.001 useKosambi=1 recombination2=0 > ./Order_LG10_xx_dist.txt

********************************************************

########################################################

# ALLMAPS - Ordering scaffolds into Chromosomes

########################################################

********************************************************

# Use the best marker order of Z to anchor scaffolds in ALLMAPS v0.8.12 (2019-05-14)

cat Order_LG10_xx_dist.txt | cut -f 1-2 | grep -v "#" | awk '{print $1, "10", $2}' | tr ' ' '\t' > Z_Allmaps.txt

# Add SNP IDs to the best order in R and create an input file to Allmaps.

> mapZ <- read.table("./Z_Allmaps.txt", header=TRUE)

> markers <- read.table("./All_SNPs_finasscont_FIL_1_LineNr.txt", header=TRUE)

> combinedZ <- merge(mapZ, markers, by="Line")

> write.table(combinedZ, "./Z_Allm.txt", sep="\t")

cat Z_Allm.txt | tr -d '"' | tr ',' '.' | cut -f 3-6 | awk '{print $3,$4,$1,$2}' | tr ' ' ',' > Z.csv

nano Z.csv # LINE-info (given and needed by Lep-MAP3 to identity SNPs) can't be kept in the output --> When you need to combine SNP+Line info, go back to the file written out from R

# Run Allmaps

source activate py27

python -m jcvi.assembly.allmaps merge Z.csv -o Map_Z.bed

python -m jcvi.assembly.allmaps path Map_Z.bed ../../../../Nicke_final_genome2.jelly.quiver.quiver.pilon_finasscont.fasta

***********************************

# Recombination rate

***********************************

### The final genetic map for Z

# Calculate genetic distance for the anchored physical marker order from Allmaps. Do this with and without PAR markers!

Z.lifted.bed # [includes 100bp gaps in the positions & correctly oriented scaffolds!]

cat Z_Allm.txt | tr -d '"' | tr ',' '.' | tr -d '*' | cut -f 2,4,5,6,7 > Z_Line.txt

# First, combine these 2 files above (new liftover physical positions and Line-numbers for SNPs (needed for Lep-MAP3)). Remove contigs without lifted positions. Leave columns: Line,Male_map,Contig,LG,Lift_phys_pos,SNP.

# Order file with physical positions and copy that order of Lines-numbers to create an order to be re-evaluated in Lep-MAP3. Save also the data combining Line-numbers and lifted phys.positions.

--> Combined_data_Z.ods

--> Order_liftPhysPos_re-evaluation.txt

# Evaluate final Z map length

java -Xmx20g -cp bin/ OrderMarkers2 evaluateOrder=./Order_liftPhysPos_re-evaluation.txt improveOrder=0 data=DATA_CALL_file_FIL_1 numThreads=11 grandparentPhase=1 minError=0.001 useKosambi=1 recombination2=0 > ./Z_distEvaluated.txt

# Add these new distance also to the file "Combined_data_Z.ods" as column "Evaluated_male_map". Save this file also to txt-format for Mareymap.

# Prepare input file to MareyMap (colums "set" "map" "mkr" "phys" "gen"). See "https://lbbe-shiny.univ-lyon1.fr/MareyMapOnline/" and article for more details on settings.

### Recombination rate estimates for 200kb bins

# For this, prepare a file including 200kb bins' start and end positions (bp)

cat chr10.357779_removed.newBins.fasta.fai.bed.200kb.MaleRecOnly.ranges.correct | cut -f 2 > Bins_200kb_toMarey.txt

nano Bins_200kb_toMarey.txt #[To add "" to header column]

# In MareyMap, fit LOESS (span 0.2) to the final Z genetic map. In "Step 4", estimate genetic positions for the bin start and end physical positions.

# Estimate genetic distance between bin start and end positions (gen.pos.of.bin_end minus gen.pos.of.bin_start)

--> LocalRecombinationRates_200kb_0.2.txt

### Recombination rate estimates for for dN/dS analyses

# Prepare a file including the midpoints (bp) of all genes. In MareyMap, fit LOESS (span of 0.1). In "Step 4", estimate the recomination rate estimate (cM/Mb) for all gene position along Z.

--> Gene_recombination_rates_0.1.txt

**Supplementary Figure 2.** One of the genome characteristics, genetic diversity, measured with alternative parameter in 200 kbp bins along the great reed warbler Z chromosome. Nucleotide diversity (pi) that was used in the manuscript (on the left) and pi adjusted with callable sites (on the right). To get more unbiased estimates of diversity, the latter was calculated based on the number of callable sites in each window. For this purpose, we used samtools depth with default settings except for specifying a minimum mapping quality of 1 to calculate coverage across the samples. We next selected sites based on the same coverage requirements as used for the variant filtering at required that at least four of the samples should have a coverage within the specified interval. From the raw set of callable sites, we used bedtools to remove overlaps with annotated repeats and to calculate the number of callable sites in each window. To get an adjusted nucleotide diversity estimate in each window we summed pi across all SNPs and divided by the number of callable sites.

**Current:** 217(-) 92(+) 134(+) 2(+) 5(-) 31(+) 98a(-)

**Without PAR:** 92(+) 134(+) 2(?) 5(+) 31(+) 98a(-)

**Supplementary Material 3**. When the PAR scaffold (217) was excluded, ALLMAPS arranged the Z scaffolds in the following order: 92(+) 134(+) 2(?) 5(+) 31(+) 98a(-). Thus, the order remained the same, but scaffold orientation changed somewhat: the orientation of scaffold 5 flipped and the orientation of scaffold 2 was left unknown. The fact that ALLMAPS cannot orient these two scaffolds with high certainty is expected as they are in the middle of the ancestral Z chromosome, i.e. in the NONREC region (see main text) where there is no recombination (i.e., genetic map is not informative). When the scaffolds were order and oriented by synteny to great tit (*Parus major*) and collared flycatcher, the most parsimonious orientation for both scaffold 2 and 5 is (+), which means that the orientation of scaffold 5 that we have used in the analysis (-) is uncertain. However, it should be noted that this uncertainty does not affect our recombination rate analyses since the entire scaffold 5 is located within the non-recombining region (NONREC), which means that its orientation in relation to neighbouring scaffolds does not affect the recombination rate estimates (see Fig. 1a).


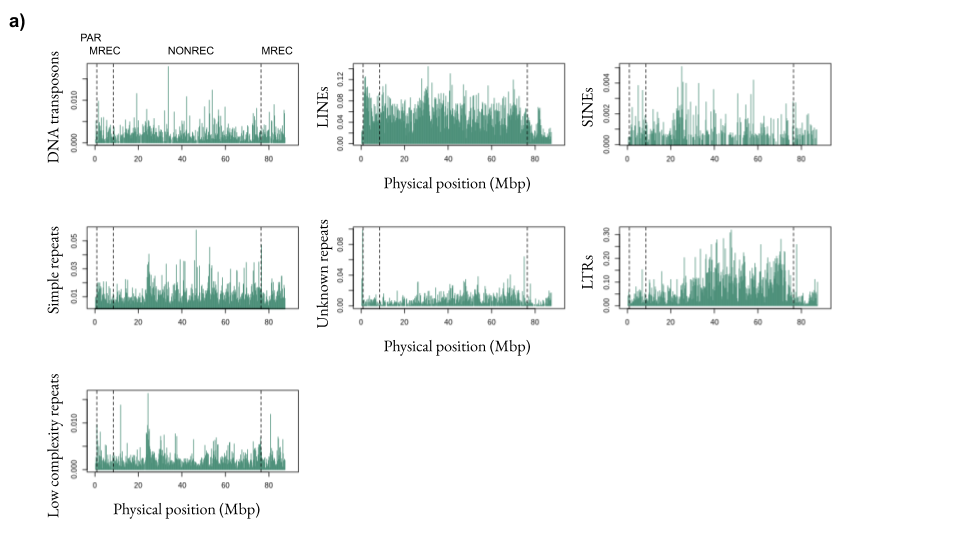

**Supplementary Figure 3**. The relationship between recombination rate and different major types of repeats (DNA transposons, LINEs, SINEs, unknown repeats, simple repeats, LTRs, and low complexity repeats) calculated as proportion of repeats in 200 kbp bins. **a)** Repeat types in relation to the physical position along the great reed warbler Z chromosome. Dashed lines mark the boundaries between the three types of recombination regions: PAR (pseudoautosomal region, where both sexes recombine), MREC (male-recombining region) and NONREC (non-recombining region). **b)** Comparison of repeat types between the three recombination regions (PAR, MREC and NONREC). Statistically significant differences (Mann-Whitney U-test, *p* < 0.05) between regions are shown and p-values that remained significant after Holm’s correction (1979) for multiple testing are marked in bold. **c)** Correlations between recombination rate (cM/200 kbp) and repeat types proportions, evaluated within ancestral- and added-Z. Correlations were tested with Kendall's *τ* and and p-values that remained significant after Holm’s correction (1979) for multiple testing are marked in bold.

**Supplementary Material 4.** Codeml generic control file:

seqfile = paml/GENE.phy * sequence data file name

treefile = tree.nwk * tree structure file name

outfile = codeml_out/GENE.mlc * main result file name

noisy = 9 * 0,1,2,3,9: how much rubbish on the screen

verbose = 1 * 0: concise; 1: detailed, 2: too much

runmode = 0 * 0: user tree; 1: semi-automatic; 2: automatic

* 3: StepwiseAddition; (4,5):PerturbationNNI; -2: pairwise

seqtype = 1 * 1:codons; 2:AAs; 3:codons-->AAs

CodonFreq = 2 * 0:1/61 each, 1:F1X4, 2:F3X4, 3:codon table

clock = 0 * 0:no clock, 1:clock; 2:local clock; 3:CombinedAnalysis

aaDist = 0 * 0:equal, +:geometric; -:linear, 1-6:G1974,Miyata,c,p,v,a

aaRatefile = dat/jones.dat * only used for aa seqs with model=empirical(_F)

* dayhoff.dat, jones.dat, wag.dat, mtmam.dat, or your own

model = 1

* models for codons:

* 0:one, 1:b, 2:2 or more dN/dS ratios for branches

* models for AAs or codon-translated AAs:

* 0:poisson, 1:proportional, 2:Empirical, 3:Empirical+F

* 6:FromCodon, 7:AAClasses, 8:REVaa_0, 9:REVaa(nr=189)

NSsites = 0 * 0:one w;1:neutral;2:selection; 3:discrete;4:freqs;

* 5:gamma;6:2gamma;7:beta;8:beta&w;9:beta&gamma;

* 10:beta&gamma+1; 11:beta&normal>1; 12:0&2normal>1;

* 13:3normal>0

icode = 0 * 0:universal code; 1:mammalian mt; 2-10:see below

Mgene = 0

* codon: 0:rates, 1:separate; 2:diff pi, 3:diff kapa, 4:all diff

* AA: 0:rates, 1:separate

fix_kappa = 0 * 1: kappa fixed, 0: kappa to be estimated

kappa = 2 * initial or fixed kappa

fix_omega = 0 * 1: omega or omega_1 fixed, 0: estimate

omega = .4 * initial or fixed omega, for codons or codon-based AAs

fix_alpha = 1 * 0: estimate gamma shape parameter; 1: fix it at alpha

alpha = 0. * initial or fixed alpha, 0:infinity (constant rate)

Malpha = 0 * different alphas for genes

ncatG = 8 * # of categories in dG of NSsites models

getSE = 0 * 0: don't want them, 1: want S.E.s of estimates

RateAncestor = 1 * (0,1,2): rates (alpha>0) or ancestral states (1 or 2)

Small_Diff = .5e-6

cleandata = 1 * remove sites with ambiguity data (1:yes, 0:no)?

* fix_blength = -1 * 0: ignore, -1: random, 1: initial, 2: fixed

method = 0 * Optimization method 0: simultaneous; 1: one branch a tim

**Supplementary Material 5.**

Results and discussion in relation to the different repeat types

In the comparison between the three recombination regions (PAR, MREC and NONREC), LINEs and LTRs were significantly higher in the NONREC compared to the PAR and MREC, whereas unknown repeats were significantly enriched in the NONREC compared to the MREC (Supplementary Figure 2b). After correction the p-value for multiple testing with Holm’s corrections (*⍺* = 0.05, *m* = 21, i.e., 7 parameters and 3 regions), comparison of LTRs between NONREC and PAR became non-significant.

When comparing the ancestral- and added-Z within the MREC, only LINEs were significantly different between the two regions (Mann-Whitney U-test: *U* = 158, *p* = 3.6e-14; higher in ancestral-Z). Correlations with recombination rate within the MREC were significant only for simple repeats (Kendall rank correlation: *τ* = 0.31, *p* = 2.4e-05). This result was observed also when tested separately for the ancestral- and added-Z (Supplementary Figure 2c). The rest of the significant correlations showed differences between the regions: LTRs were positively correlated in the added-Z and negatively in the ancestral-Z. Unknown repeats had a positive correlation with recombination rate only in the added-Z and LINEs only in the ancestral-Z (Supplementary Figure 2c). After correction the p-value for multiple testing with Holm’s corrections (*⍺* = 0.05, *m* = 7, i.e., 7 parameters in both ancestral- and added-Z), LTRs correlation in added-Z and LTRs and SRs correlations in ancestral-Z became non-significant.

Associations between different repeat types and recombination showed complex results. LTRs had the expected pattern with recombination, as they were accumulated in the NONREC. Region comparison supported accumulation of LINEs in the absence of recombination too, but when correlated with the rate within the MREC, ancestral-Z showed positive association. Thus, only some types of repeats seem to accumulate in the absence of recombination in the great reed warbler Z. The observed positive correlations are in line with earlier observation in birds, where association between recombination rate and transposable elements was suggested to reflect an indirect relationship between retrotransposons and recombination due to the shared preference for an accessible chromatin state (Kawakami et al., 2017).

**References**

Bao, W., Kojima, K. K., & Kohany, O. (2015). Repbase Update, a database of repetitive elements in eukaryotic genomes. *Mobile DNA,* 6:11.

Hansson, B., Sigeman, H., Stervander, M., Tarka, M., Ponnikas, S., Strandh, M., … & Hasselquist, D. (2018). Contrasting results from GWAS and QTL mapping on wing length in great reed warblers. *Molecular Ecology Resources*, *18*, 867–876.

Hillier, L. W., Miller, W., Birney, E., Warren, W., Hardison, R. C., Ponting, C. P., Bork, P., Burt, D. W., Groenen, M. A. M., Delany, M. E., et al. (2004). Sequence and comparative analysis of the chicken genome provide unique perspectives on vertebrate evolution. *Nature, 432,* 695–716.

Danecek, P., Auton, A., Abecasis, G., Albers, C. A., Banks, E., DePristo, … & 1000 Genomes Project Analysis Group (2011). The variant call format and VCFtools. *Bioinformatics*, *27*, 2156–2158.

Garrison, E. & Marth, G. (2012). Haplotype-based variant detection from short-read sequencing. Preprint at<https://arxiv.org/abs/1207.3907>.

Holm, S. (1979). A simple sequentially rejective multiple test procedure. *Scandinavian Journal of Statistics,* 6, 65–70.

Kawakami, T., Smeds, L., Backström, N., Husby, A., Qvarnström, A., Mugal, C. F., … & Ellegren, H. (2014). A high-density linkage map enables a second-generation collared flycatcher genome assembly and reveals the patterns of avian recombination rate variation and chromosomal evolution. *Molecular ecology*, 23, 4035–4058.

Puritz, J. B., Hollenbeck, C. M., & Gold, J. R. (2014). dDocent: a RADseq, variant-calling pipeline designed for population genomics of non-model organisms. *PeerJ* 2:e431

Suh, A., Smeds, L., & Ellegren, H. (2018). Abundant Recent Activity of Retrovirus-like Retrotransposons within and among Flycatcher Species Implies a Rich Source of Structural Variation in Songbird Genomes. *Molecular Ecology*, *27*, 99–111.

Sigeman, H., Strandh, M., Proux-Wéra, E., Kutschera, V. E., Soler, S., Ponnikas, S., … & Hansson B. (2020b). Genomics of an avian neo-sex chromosome reveals the evolutionary dynamics of recombination suppression and sex-linked genes. *bioRxiv*, 2020.09.25.314088; doi: https://doi.org/10.1101/2020.09.25.314088

Vijay, N., Bossu, C. M., Poelstra, J. W., Weissensteiner, M. H., Suh, A., Kryukov, A. P., & Wolf, J. B. W. (2016). Evolution of heterogeneous genome differentiation across multiple contact zones in a crow species complex. *Nature Communications, 7*, 13195.

Warren, W. C., Clayton, D. F., Ellegren, H., Arnold, A. P., Hillier, L. W., Künstner, A., … & Wilson, R. K. (2010). The genome of a songbird. *Nature, 464*, 757–762.
